# Trastuzumab-Mediated Antibody-Dependent Cell-Mediated Cytotoxicity (ADCC) Enhances Natural Killer Cell Cytotoxicity in HER2-Overexpressing Ovarian Cancer

**DOI:** 10.3390/ijms252111733

**Published:** 2024-10-31

**Authors:** Sa Deok Hong, Nar Bahadur Katuwal, Min Sil Kang, Mithun Ghosh, Seong Min Park, Tae Hoen Kim, Young Seok Baek, Seung Ryeol Lee, Yong Wha Moon

**Affiliations:** 1 Department of Biomedical Science, The Graduate School, CHA University, Seongnam-si 13488, Republic of Korea; 2 Department of Pathology, CHA Bundang Medical Center, CHA University, Seongnam-si 13496, Republic of Korea; 3 Immunotherapy Team, New Biological Entity (NBE) Research, R&D Division, CHA Biotech, Seongnam-si 13488, Republic of Korea; 4 Department of Urology, CHA Bundang Medical Center, CHA University, Seongnam-si 13496, Republic of Korea; 5 Division of Hematology-Oncology, Department of Internal Medicine, CHA Bundang Medical Center, CHA University, Seongnam-si 13496, Republic of Korea

**Keywords:** HER2-overexpressed ovarian cancer, trastuzumab, NK cell, ADCC

## Abstract

Ovarian cancer is the deadliest gynecologic cancer. Although human epidermal growth factor receptor-2 (HER2) overexpression, a poor prognostic molecular marker in ovarian cancer, is found in almost 30% of ovarian cancer cases, there are no established therapies for HER2-overexpressing ovarian cancer. In this study, we investigated the efficacy of combined samfenet, a biosimilar compound of trastuzumab, and natural killer (NK) cells in preclinical model of HER2-overexpressing ovarian cancer. Firstly, we screened the HER2 expression in three ovarian cancer cell lines and eight ovarian cancer patient-derived tumor xenograft (PDTX) samples. Then, immunohistochemistry and silver in situ hybridization (SISH) were performed following clinical criteria. HER2-overexpressing cells exhibited the highest sensitivity to samfenet compared with low-HER2-expressing cells. In addition, the combination of samfenet with natural killer (NK) cells resulted in significantly enhanced sensitivity to HER2-overexpressing cells and showed a significant antitumor effect on PDTX mice compared with monotherapy. It is known that anti-HER2-humanized IgG1 monoclonal antibodies, including trastuzumab, induce antibody-dependent cellular cytotoxicity (ADCC). Consequently, the combination of samfenet with NK cells demonstrated NK cell-mediated ADCC, as confirmed using an in vitro NK cytotoxicity assay and in vivo antitumor efficacy. A transferase dUTP nick end labeling (TUNEL) assay using xenografted tumors further supported the ADCC effects based on the increase in the number of apoptotic cells in the combination group. Furthermore, high HER2 expression was associated with shorter progression-free survival and overall survival based on public mRNA expression data. In this study, we demonstrated that the combination of samfenet and NK cell therapy could be a promising treatment strategy for patients with HER2-overexpressing ovarian cancer, through ADCC effects. Therefore, this study supports a rationale for further clinical studies of the combination of samfenet and NK cells as a therapy for patients with HER2-overexpressing ovarian cancer.

## 1. Introduction

Ovarian cancer is a leading cause of cancer-related mortality among women in developed countries, and it is considered one of the deadliest gynecologic cancers [1]. Ovarian cancers arise from the ovarian surface epithelium, peritoneum and the fallopian tube [2], based on histopathology and molecular genetics alterations, and are classified into five major types: high-grade serous carcinoma (68% of all cases), clear cell carcinoma (12%), endometrioid carcinoma (11%), mucinous carcinoma (3%), and low-grade serous carcinoma (3%) [1,3].

Despite the continuous efforts of numerous clinicians globally, the prognosis of ovarian cancer has remained poor over the past several decades because of the delayed detection and early recurrence after the initial response to primary platinum-based chemotherapy [4]. Currently, the recommended first-line therapy includes surgery followed by chemotherapy based on a combination of a platinum drug and paclitaxel or neoadjuvant chemotherapy with the same regimen, followed by interval debulking surgery [5]. Although systemic anticancer therapies, including the use of poly (ADP-ribose) polymerase (PARP) inhibitors [6,7,8] and bevacizumab [9], have recently shown progress in the maintenance setting of ovarian cancer, their efficacies are very limited in chemotherapy-resistant recurrent ovarian cancer. In addition, several preclinical and clinical studies are ongoing for the treatment of recurrent ovarian cancer using immunotherapy, which is currently not approved in recurrent ovarian cancer [10]. Therefore, the development of new therapies remains an unmet medical need.

Human epidermal growth factor receptor-2 (HER2) is one of a family of four membrane tyrosine kinases that mediates cell growth, differentiation, and survival [11]. Although HER2 is associated with poor prognosis in breast cancer, its role in ovarian cancer remains controversial [4]. Trastuzumab (Herceptin^®^, Basel, Switzerland), a humanized monoclonal antibody (mAb) that targets HER2, promotes HER2 internalization and downregulation. At the beginning of trastuzumab development, it was approved for the treatment of HER2-overexpressing breast carcinomas, prompting its approval for the treatment of patients with HER2-positive metastatic gastric cancer [12]. Furthermore, various preclinical and clinical trials are underway to expand trastuzumab indications in other solid cancer types [13]. However, its effectiveness against ovarian cancer remains to be properly established [4]. In a meta-analysis of HER2 expression in ovarian cancer, which included 34 studies with 5180 patients, HER2 expression correlated negatively with progression-free survival (PFS), disease-free survival, and overall survival (OS) [14]. Therefore, previous studies have attempted to use lapatinib (HER2 tyrosine kinase inhibitor), pertuzumab or trastuzumab (HER2 dimerization inhibitors), and trastuzumab emtansine or trastuzumab deruxtecan (HER2 monoclonal antibody–drug conjugates) to treat patients with HER2-expressing ovarian cancer [15].

As an antibody, trastuzumab blocks HER2 signaling and recruits cytotoxic effector cells via the Fcγ part of the IgG1 antibody, consequently triggering antibody-dependent cellular cytotoxicity (ADCC), which can involve granulocytes, monocytes, macrophages, dendritic cells, and natural killer (NK) cells [11,16,17,18]. Furthermore, CD16-expressing NK cells are mainly associated with major ADCC mediators [19]. A previous preclinical study demonstrated that trastuzumab reduced tumor volume by 96% in NK cell-competent nude mice compared with CD16 knockout mice [20]. Furthermore, a phase II clinical trial of allogeneic NK cells with recombinant human interleukin-12 in 14 patients with recurrent ovarian cancer showed a 21.4% partial response rate (NCT00652899). However, other clinical trials exhibited poor prognosis following NK cell administration [21]. Moreover, NCT05395052 studied treatment of autologous NK cells combined with trastuzumab in advanced solid cancer, but the study was terminated.

Based on the absence of therapeutic options for HER2-overexpressing ovarian cancer, this study aimed to determine the anticancer efficacy of samfenet, a biosimilar compound of trastuzumab, combined with NK cells in preclinical models. It demonstrated that the combination of samfenet with NK cells exhibited a synergistic ADCC effect and significantly inhibited a HER2-overexpressing ovarian cancer patient-derived tumor xenograft (PDTX) in vivo model. This study provides a scientific rationale for further clinical development of this combination therapy in HER2-overexpressing ovarian cancer.

## 2. Results

### 2.1. Screening for HER2 Expression in Ovarian Cancer Cell Lines

**Figure 1:**
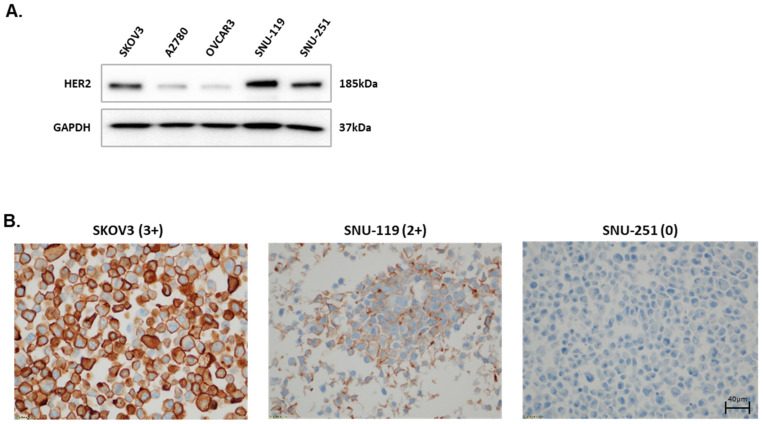
(**A**) Immunoblot showing HER2 expression in ovarian cancer cell lines. (**B**) Immunohistochemistry for human HER2 in ovarian cancer cell blocks. Staining images were taken at 200× magnification.

For patients with HER2-positive metastatic breast cancer, trastuzumab, an FDA-approved mAb targeting HER2, showed significant clinical benefits in both metastatic and adjuvant settings [22]. However, despite HER2 expression in ovarian cancer, its efficacy is limited [23]. In this study, we analyzed the efficacy of samfenet, a biosimilar compound of trastuzumab, combined with NK cells using ovarian cancer preclinical models. To determine whether samfenet is effective in the HER2-positive ovarian cancer preclinical model, HER2 expression in various ovarian cancer cell lines, including SKOV3, A2780, OVCAR3, SNU-119, and SNU-251, was assessed. Western blotting showed that SKOV3, SNU-119, and SNU-251 cells had higher HER2 expression than A2780 and OVCAR3 cells (Figure 1A).

To further validate HER2 expression following clinical criteria, immunohistochemistry (IHC) was performed using HER2-expressing cell lines (SKOV3, SNU-119, and SNU-251). The data confirmed that SKOV3 demonstrated the highest HER2 expression (HER2, 3+), followed by SNU-119 (HER2 2+) and SNU-251 (HER2-negative) (Figure 1B). In SNU-251 cells, the HER2 Western blot band revealed high expression compared with the IHC score, which indicates the reduced sensitivity of IHC relative to the Western blot band [24]. In detail, the Western blot technique included a step for lysing the cell, which facilitates efficient protein extraction and preserved antibody recognition sites on proteins. Furthermore, the separation of proteins based on size, charge, and conformation using gel electrophoresis increased detection sensitivity [25]. However, IHC included a step for fixation, which could mask epitopes or affect protein conformation, decreasing the detection sensitivity and potentially leading to false negatives [26,27].

### 2.2. Samfenet Anticancer Efficacy and NK Cell-Mediated Antibody-Dependent Cellular Cytotoxicity Efficacy Test

**Figure 2:**
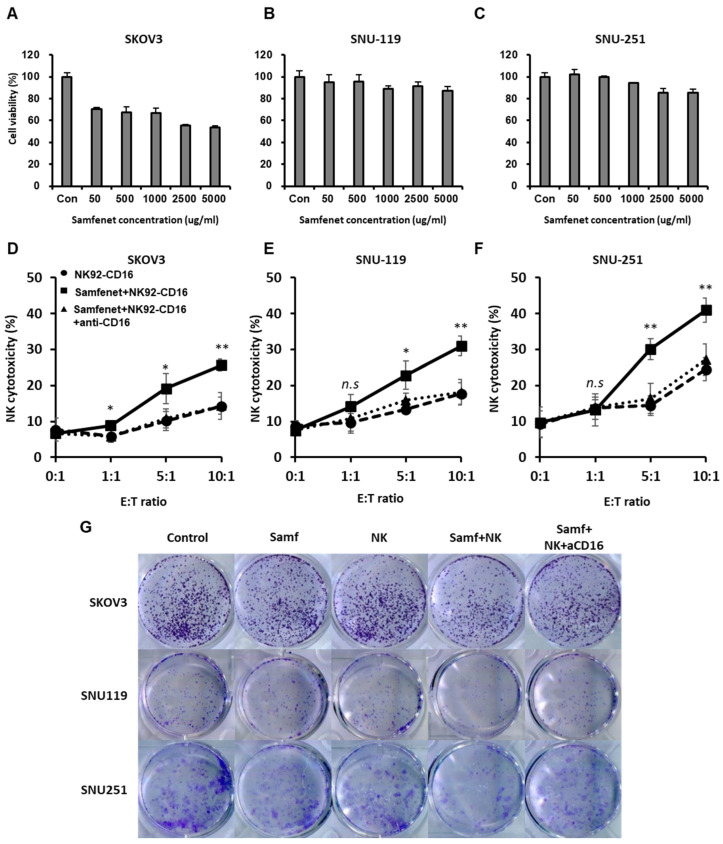
(**A**–**C**) Cell viability (MTT) assays of (**A**) SKOV3, (**B**) SNU-119, and (**C**) SNU-251 cell lines treated with various samfenet concentrations for 72 h. Three independently repeated experiments were performed with similar results. (**D**–**E**) The NK cytotoxicity assay in the presence or absence of samfenet was analyzed using CFSE-7AAD assay. Target cells [(**D**) SKOV3, (**E**) SNU-119, and (**F**) SNU-251] and effector cells (NK92-CD16 cells) were co-cultured at 1:1, 5:1, and 10:1 E/T ratios for 4 h with 5 mg/mL of samfenet. Three independently repeated experiments were performed with similar results. Error bars represent the standard deviations of three independent experiments. Student’s *t*-test between NK92-CD16 and Samfenet+NK92-CD16: * *p* < 0.05, ** *p* < 0.01. Abbreviation: ns, not significant. (**G**) Colony formation assay in the presence or absence of samfenet and NK92-CD16 with or without CD16 blocking. Target cells (SKOV3, SNU-119, SNU-251) and effector cells (NK92-CD16 cells) were co-cultured at 10:1 E/T ratio for 4 h with 400 ug/mL of samfenet. The photographs were taken 10 days after culture. Abbreviation: Samf: Samfenet; NK: NK92-CD16; aCD16: CD16 antibody.

Based on the HER2 expression in ovarian cancer cell lines, we sought to analyze the anticancer efficacy of samfenet, which could be effective in treating HER2-expressing ovarian carcinoma. First, the sensitivity of samfenet in the SKOV3, SNU-119, and SNU-251 cell lines was determined, with the highest HER2-expressing cell line, SKOV3, showing the highest sensitivity compared with the low-HER2-expressing cell lines, SNU-119 and SNU-251 (Figure 2A–C). However, the efficacies of samfenet in SNU-251 and SNU-119 cell lines were similar despite the varied HER2 expression, possibly because of the mAb-based therapy, which interferes with the HER2 receptor [28].

Previous studies have demonstrated that NK cell-mediated ADCC plays an important role in anti-HER2 antibody therapy [29]. Therefore, enhancement of NK cell-mediated ADCC could be the better approach to improve the anticancer efficacy of samfenet [30,31]. Consequently, the ADCC effect was analyzed using an NK cytotoxicity assay. The NK cells were co-cultured with samfenet-pretreated ovarian cancer cells (SKOV3, SNU119, and SNU 251) at various E/T ratios (NK/cancer cell) of 1:1, 5:1, and 10:1, which significantly enhanced NK cytotoxicity compared with the monotherapy and control groups (Figure 2D–F). Expectedly, CD16 blocking significantly decreased NK cytotoxicity because of NK Fc receptor blocking [32] (Figure 2D–F). Additionally, we performed a colony formation assay to evaluate how the NK cell-mediated ADCC affects cancer cell proliferation. The NK cells were co-cultured with samfenet-treated ovarian cancer cells (SKOV3, SNU119, and SNU 251) in a 10:1 ratio (NK/cancer cell), which significantly decreased cancer cell proliferation compared with monotherapy and control groups. Furthermore, CD16 blocking significantly increased cancer cell proliferation (Figure 2G).

### 2.3. Screening of HER2-Overexpressing Patient-Derived Tumor Xenograft Models

**Figure 3:**
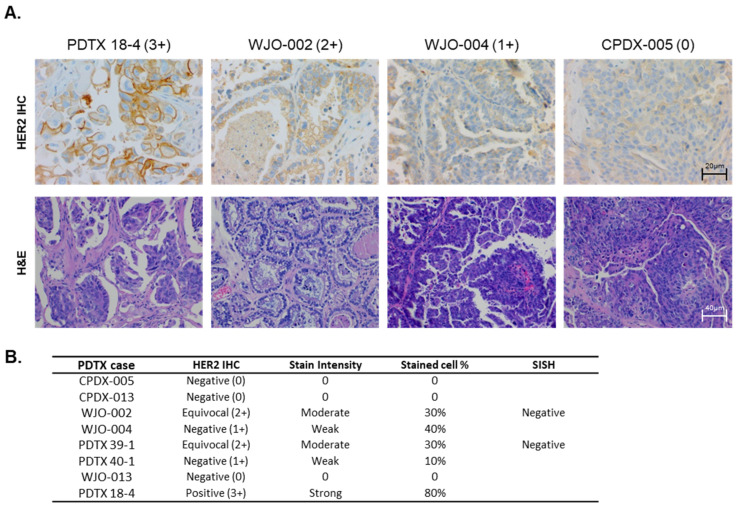
(**A**) Representative immunohistochemistry staining of human HER2 in PDTX cases. Staining images were collected at 400× magnification (HER2 IHC) and 200× magnification (H&E). HER2 expression was scored as positive (3+), equivocal (2+), or negative (1+ and 0) as indicated. (**B**) HER2 protein expression levels in ovarian cancer PDTX cases. First, HER2 expression was examined using immunohistochemistry, and equivocal cases (HER2 IHC 2+) were further analyzed using SISH.

After demonstrating the significant ADCC effect on ovarian cancer cell lines, HER2 expression was further evaluated in eight ovarian cancer patient-derived tumor xenograft (PDTX) models. Figure 3A presents the representative images of HER2 IHC ranging from HER2 0 to 3+. PDTX 18-4 showed HER2 3+, that is HER2-overexpressing, WJO-002 showed HER2 2+, that is HER2-equivocal, and WJO-004 and CPDX-005 showed HER2 1+/0, that is HER2-negative (Figure 3A). We further performed the silver in situ hybridization (SISH) to identify HER2-equivocal PDTX cases with HER2 gene amplification. WJO-002 and PDTX 39-1 did not amplify the HER2 gene. Figure 3B presents a summary of the level of HER2 expression in eight ovarian cancer PDTX models.

### 2.4. Combined Samfenet and NK Cell Therapy Synergistically Regresses the HER2-Overexpressing PDTX Models

**Figure 4:**
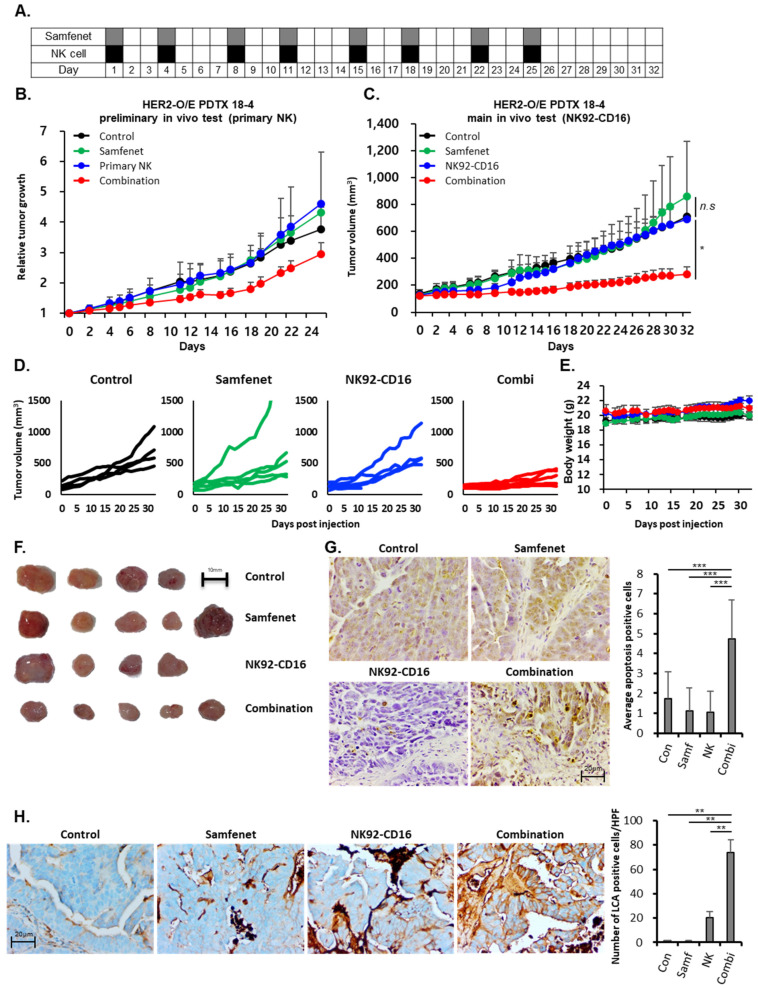
(**A**) Treatment schedule for main in vivo efficacy testing using HER2-overexpressing PDTX model (PDTX 18-4). (**B**) The average relative tumor growth of HER2-overexpressing PDTX 18-4 tumors, which were treated with indicated drugs, in the preliminary in vivo test using primary NK cells. Error bars represent the standard error of the mean (SEM) of two tumors per group. (**C**) The average tumor volume of HER2-overexpressing PDTX 18-4 tumors, which were treated with indicated drugs, in the main in vivo test using NK92-CD16 cells. Error bars represent the SEM of five tumors per group. Student’s *t*-test: * *p* < 0.05. Abbreviation: ns, not significant. (**D**) Tumor growth curves of individual mice in the indicated treatment groups of the HER2-overexpressing PDTX 18-4 model. (**E**) Average body weight of mice with indicated groups. Error bars represent the standard deviations of five mice per group. (**F**) Gross harvested tumor. (**G**) TUNEL assay using PDTX 18-4 xenografted tumor after sacrifice. Staining images per group are shown, and bar graphs represent the average number of apoptotic cells per group in five random, non-overlapping fields at 400× magnification. Data are presented as mean ± SD. Student’s *t*-test: *** *p* < 0.001. Abbreviation: Con: control; Samf: samfenet; NK: NK92-CD16; Combi: combination. (**H**) Immunohistochemistry for human leukocyte common antigen (LCA) using PDTX 18-4 xenografted tumor after sacrifice. Staining images per group are shown, and bar graph represents the average number of LCA-positive cells per group in five random, non-overlapping fields at 400× magnification. Data are presented as mean ± SD. Student’s *t*-test: ** *p* < 0.01. Abbreviation: Con: control; Samf: samfenet; NK: NK92-CD16; Combi: combination.

Based on our previous IHC experiments, we confirmed that PDTX 18-4 is the highest HER2-overexpressing PDTX model exhibiting HER2 3+. Using the PDTX 18-4 model, the antitumor efficacy of samfenet combined with primary NK cells was tested. First, in a preliminary experiment, a small number of mice (two per group) were used. The feasibility and antitumor efficacy of samfenet, primary NK cells that express the CD16 receptor, and a combination of both were analyzed (Figure 4B). After confirming that the combination of samfenet and primary NK cells was feasible in the preliminary experiment, the antitumor efficacy of samfenet, NK92-CD16, and the combination of both in another set of mice (five per group) was further tested.

As mentioned in the Section 4, when the HER2-overexpressing PDTX tumor reached 80–100 mm^3^, 10 mg/kg of samfenet was administered intraperitoneally (IP) twice weekly, and 1 × 10^7^ primary NK cells or 5 × 10^6^ NK92-CD16 cells were administered intravenously (IV) twice weekly in parallel with samfenet injection. For the combination group, samfenet was first injected, followed by NK cell injection. Primary NK in vivo experiments were performed for 24 days (six injections total) and NK92-CD16 (eight injections total) in vivo experiments for 32 days.

This study showed that the combination of samfenet and primary NK cells effectively inhibited tumor growth compared with monotherapy (Figure 4B). Similarly, the combination of samfenet and NK92-CD16 significantly reduced the tumor size compared with NK92-CD16 monotherapy (*p* = 0.027) and the control group (*p* = 0.016) (Figure 4C). Figure 4D shows the individual tumor volumes. After 32 days of drug treatment, mice were sacrificed, and the tumors were excised. Figure 4E shows the photographs of excised tumors. A further terminal deoxynucleotidyl transferase dUTP nick end labeling (TUNEL) assay was performed using the excised tumors. The TUNEL assay results demonstrated increased apoptosis in the combination group compared with the samfenet (*p* < 0.001) and NK92-CD16 (*p* = 0.001) monotherapy groups, further supporting the results of tumor growth inhibition.

Infiltration of NK cells in tumors is a significant factor that can be used to evaluate the anticancer effect of adoptive NK transfer [33]. Moreover, patients who exhibited an increased clinical response to mAb-based therapy showed enhanced NK cell infiltration [31,34]. Naturally, both the control and samfenet monotherapy groups did not show any NK cell infiltration as we did not inject NK cells. Notably, NK cell infiltration was significantly increased in the combination of samfenet and NK92-CD16 compared to NK92-CD16 monotherapy (Figure 4H).

### 2.5. High HER2 Expression Is Associated with Poor Prognosis in Patients with Ovarian Cancer

**Figure 5:**
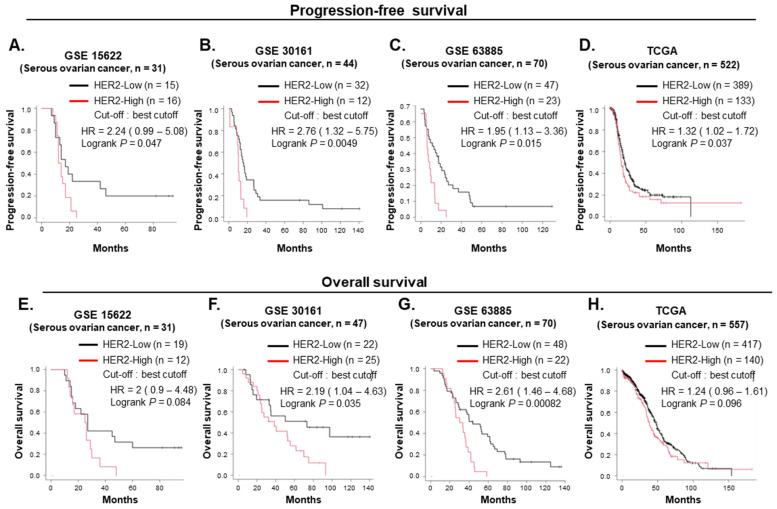
(**A**–**D**) Kaplan–Meier survival curves of progression-free survival in serous-histology ovarian cancer according to HER2 mRNA expression from public mRNA microarray datasets of (**A**) GSE 15622, (**B**) GSE 30161, (**C**) GSE 63885, and (**D**) TCGA. Data were analyzed using the online platform Kaplan–Meier plotter (www.kmplot.com, accessed on 30 June 2024). (**E**–**H**) Kaplan–Meier survival curves of overall survival in serous-histology ovarian cancer according to HER2 mRNA expression from public mRNA microarray datasets of (**E**) GSE 15622, (**F**) GSE 30161, (**G**) GSE 63885, and (**H**) TCGA. Data were analyzed using the online platform Kaplan–Meier plotter (www.kmplot.com, accessed on 30 June 2024).

We investigated the prognosis of HER2 expression in patients with serous-histology ovarian cancer, which is a major subtype of ovarian cancer, using independent public mRNA expression datasets. The cutoffs were set to auto-select the best cutoff for GSE 15622, GSE 30161, GSE 63885, and The Cancer Genome Atlas (TCGA). Expectedly, the patient group showing high HER2 expression (HER2-High group) had shorter PFS and OS compared with the low-HER2-expressing (HER2-Low group) patients with serous-histology ovarian cancer (Figure 5A–H). Taken together, these findings based on a public mRNA database, showed that high HER2 expression was associated with poor prognosis in patients with serous ovarian cancer.

## 3. Discussion

This study aimed to address the unmet need for effective HER2-targeting therapies in HER2-overexpressing ovarian cancer by analyzing HER2 expression in ovarian cancer cell lines and PDTX models. Although several HER2-targeting agents, including trastuzumab and its biosimilar compounds, such as samfenet and herzuma, are FDA-approved for HER2-overexpressing breast and gastric cancers, no such agents have been approved for HER2-overexpressing ovarian cancer [19,35,36]. For the first time, we evaluated the efficacy of samfenet combined with CD16-expressing NK cells in ovarian cancer models. Our findings demonstrated that the combination of samfenet and NK cells significantly enhanced ADCC in vitro and markedly reduced tumor size in vivo, suggesting a promising therapeutic strategy for patients with this subset of ovarian cancer.

Trastuzumab, an anti-HER2 mAb, is associated with both non-immune and immune-mediated mechanisms of action. In non-immune-related mechanisms, its fragment antigen-binding (Fab) domain directly binds to the HER2 receptors on the tumor cell surface and inhibits downstream signaling, leading to antiproliferative effects [37]. For immune-related mechanisms, the fragment crystallizable (Fc) portion is recognized by Fc receptors, which are expressed by NK cells, leading to NK cell activation [37,38]. These activated NK cells then release cytotoxic granules containing perforins and granzymes that induce cell death, which is called ADCC [39].

In this study, the efficacy of samfenet in HER2-expressing SKOV3, SNU-119, and SNU-251 cells was first evaluated by analyzing cell viability. It was found that one of the highest HER2-expressing cells, SKOV3, showed the highest cytotoxicity to the cells compared with the low-HER2-expressing cell lines, SNU-119 and SNU-251. This result indicates that samfenet is effective against HER2-expressing ovarian cancer. Furthermore, several studies have demonstrated that trastuzumab induces ADCC effects [40,41,42]. Then, the samfenet-pretreated cells were co-cultured with NK cells. NK cell-mediated killing or ADCC effect was observed in the SKOV3, SNU-119, and SNU-251 cell lines. Moreover, our in vivo study using a HER2-overexpressing PDTX model demonstrated that combining samfenet with an injection of NK92-CD16 cells significantly reduced tumor size compared with monotherapy, confirming the NK cell-mediated ADCC effect.

As mentioned above, ADCC effect has been studied in various research. Among these studies, there is research that focused on enhancing the trastuzumab-mediated ADCC effect in HER2-expressing tumors. Du et al. revealed that podoplanin-positive cancer-associated fibroblasts secreted ADCC-suppressing factors, indoleamine 2,3-dioxygenase 1 (IDO1) and tryptophan 2,3-dioxygenase 2 (TDO2). A dual inhibitor targeting IDO1/TDO2 enhanced the ADCC effect [43]. Another study demonstrated that the downregulation of intracellular adhesion molecule 1 (ICAM-1) enabled HER2-expressing cancer cells to escape the ADCC effect. However, HER2-engineered CAR NK cells overcame the ICAM-1-mediated ADCC inhibition [44]. Furthermore, NK cell priming therapy, which increased the recruitment of NK cells to tumors, enhanced the efficacy of NK therapy. DF1001, a tri-specific NK cell engager which carries NK Group 2D (NKG2D), CD16, and HER2, effectively treated patients with advanced solid tumors (NCT04143711). Another tri-specific NK engager, TriKE, formed of IL-15, CD16, and mesothelin, showed potential to overcome the immunosuppressive TME of NSCLC patients [45]. Although the newly developed drugs show good anticancer effects, they are still in the early stages of development. Therefore, it takes a long time to gain approval in a clinical setting. A key advantage of our study is that it utilizes already developed trastuzumab and allogeneic NK cell therapies, which allows for faster implementation of combination clinical trials compared to other studies.

In gynecologic cancers, amplification or overexpression of the HER2 oncogene plays an important role in carcinogenesis. HER2 overexpression is highly variable in ovarian cancer, ranging from 2% to 66%, although the efficacy of HER2-targeted therapy remains controversial [46]. Numerous clinical trials on HER2-targeted therapy for patients with HER2-expressing ovarian cancer are currently ongoing, and several trials have already been completed (Table S1). More importantly, in a phase II clinical trial (NCT00189579), 41 patients with HER2-overexpressing ovarian cancer who were treated with paclitaxel and carboplatin alone or in combination with trastuzumab showed modest activity, with an objective response rate of 7.3% [47]. Furthermore, a phase II clinical trial (NCT04639219) of trastuzumab deruxtecan in 102 patients with solid tumors with HER2-activating mutant cancer showed an objective response rate of 29.4% according to an independent central review. This study showed the antitumor activity and durable responses in heavily pretreated patients across multiple tumor types with activating HER2 mutations [48]. However, few clinical trials have shown the efficacy against HER2-expressing ovarian cancer. To enhance the anticancer effect of HER2-targeted therapy, a novel combination is required. Accordingly, NK cell therapy could be considered as a trastuzumab combinator on the basis of the potential ADCC effect. A phase I trial involving allogeneic NK cells combined with trastuzumab is being conducted in advanced HER2-expressing solid cancers (NCT05395052). A clinical trial of NK cells combined with trastuzumab for treating patients with HER2-overexpressing ovarian cancer should be conducted based on our preclinical study.

To speculate the potential toxicities of the combination of NK cells with samfenet, we searched for publications regarding the toxicities of combined NK cell and anti-HER2 therapies but found none. Generally, trastuzumab is well tolerated in patients with breast cancer, except for cardiac toxicities in some patients [49]. NK cell therapy is often used to treat various types of cancer, including lymphoma, leukemia, and solid tumors [50]. Common toxicities of NK cell therapy include asthenia, fatigue, anorexia, and chills, which are mild and manageable [51,52]. Furthermore, in an animal experiment, the combination of NK cells and samfenet did not cause weight loss, suggesting the absence of systemic toxicity. However, when conducting a clinical trial using this combination, toxicities, including immune-mediated or cardiac toxicities, should be closely monitored.

In conclusion, this study demonstrated that the combination of samfenet, a biosimilar compound of trastuzumab, and NK cell therapy showed remarkable antitumor efficacy in a preclinical model of HER2-overexpressing ovarian cancer through the ADCC effect. There are no established therapies for HER2-overexpressing ovarian cancer. Therefore, this study supports a rationale for further clinical development of combined NK cell and samfenet therapy.

## 4. Materials and Methods

### 4.1. Drugs and Cell Lines

Samfenet was provided by the Daewoong Pharmaceutical Company (Seoul, Republic of Korea). Samfenet was dissolved in phosphate-buffered saline (PBS) for in vitro and in vivo studies. Primary NK (pNK) cells were provided by the CHA Biotech company (Seongnam, Republic of Korea). CD16-expressing (NK92-CD16) NK cells were kindly supplied by Semi Kim from Korea University, who purchased cells from the American Type Culture Collection (Manassas, VA, USA). The ovarian cancer cell lines (A2780, OVCAR3, and SKOV3) were purchased from the American Type Culture Collection (Manassas, VA, USA). The other ovarian cancer cell lines (SNU-119 and SNU-251) were purchased from the Korean Cell Line Bank (Seoul, Republic of Korea).

### 4.2. Cell Culture

The NK92-CD16 cell line was cultured in alpha minimum essential medium (Welgene Inc., Daegu, Republic of Korea) containing 12.5% heat-inactivated fetal bovine serum (FBS) (Welgene Inc., Daegu, Republic of Korea), 12.5% horse serum (ThermoFisher Scientific, Waltham, MA, USA), recombinant human interleukin-2 (IL-2) 500 IU IL-2/mL (Peprotech, Rocky Hill, NJ, USA), and 1% penicillin/streptomycin (Welgene Inc., Daegu, Republic of Korea). The SKOV3, A2780, OVCAR3, SNU-119, and SNU-251 cell lines were cultured in RPMI 1640 medium (Welgene Inc., Daegu, Republic of Korea) containing 10% heat-inactivated FBS (Welgene Inc., Daegu, Republic of Korea) and 1% penicillin/streptomycin solution (Welgene Inc., Daegu, Republic of Korea). All cells were maintained in a humidified atmosphere containing 5% CO_2_ at 37 °C. All cells were confirmed to be free of mycoplasma and cultured for up to 1 month to ensure their authenticity.

### 4.3. Western Blotting

Cell proteins were extracted using radioimmunoprecipitation lysis buffer (cat# 89901, ThermoFisher Scientific, Waltham, MA, USA) with the addition of a protease inhibitor cocktail (cat# 11873580001, Roche, Germany) and phosphatase inhibitors (cat# 1862495, ThermoFisher Scientific, Waltham, MA, USA). The isolated protein concentration was measured using the Pierce™ BCA protein assay kit (cat# 23228 and cat# 1859078, ThermoFisher Scientific, Waltham, MA, USA). Western blotting was performed as previously described [53]. The primary antibodies used for Western blot were HER2 (ab134182, Abcam) or glyceraldehyde 3-phosphate dehydrogenase (cat# 2118, Cell signaling), and the secondary antibodies were anti-mouse horseradish peroxidase (HRP) (cat# GTX213111-01, GeneTex) or anti-rabbit HRP (cat# GTX213110-01, GeneTex).

### 4.4. Immunohistochemistry

Approximately 1 × 10^7^ cells were harvested from each cell line (SKOV3, SNU-119, and SNU-251). Subsequently, cell blocks were prepared using agarose gel (Y50004, Young Science). PDTX blocks were also prepared. IHC was conducted on cell and tissue blocks to analyze HER2 expression.

IHC was performed as previously described [54]. Briefly, cell block and PDTX tissues were deparaffinized, subjected to an antigen retrieval process, stained with human leukocyte common antigen (LCA) or HER2 primary antibody at a 1:200 dilution (ab134182, Abcam), and then incubated overnight at 4 °C. The next day, sections were washed, and the HRP-conjugated secondary antibody was incubated for 1 h at room temperature. Sections were counterstained with hematoxylin, and positively stained cells were observed under a light microscope at 400× microscopic fields. ImageJ software version 1.53t was used to quantify positively stained cells.

The HER2 immunostaining results were categorized as previously described [55]. Briefly, tumors with complete absence of staining in <10% of the tumor cells were scored as 0; tumors with faint membrane staining along with some membrane staining in >10% of the tumor cells were scored as 1+; tumors with weak-to-moderate complete membrane staining in >10% of the tumor cells were scored as 2+; and tumors with strong complete membrane staining in >10% of the tumor cells were scored as 3+.

### 4.5. Silver In Situ Hybridization Assay

HER2 SISH assays were processed using Ventana’s Benchmark ULTRA automated slide stainers. The VENTANA HER2 Dual ISH DNA Probe Cocktail was used to determine HER2 gene amplification by measuring the HER2 gene-to-Chromosome 17 (Chr 17) ratio under light microscopy [56]. Results were determined following the ASCO/CAP guidelines.

For HER2 and Chr 17 SISH assay processing, Ventana’s Benchmark ULTRA automated slide stainers were used. Automated SISH of consecutive slides from the same paraffin blocks used for hematoxylin and eosin staining were performed for INFORM HER2 DNA and Chr 17 probes. Both probes were labeled with dinitrophenol and optimally developed for use with the ultraVIEW SISH detection kit and accessory reagents on the Ventana’s Benchmark series of automated slide stainers. The HER2 DNA probe was denatured at 95 °C for 12 min and hybridized at 52 °C for 2 h. After hybridization, appropriate stringency washes (three times at 72 °C) were performed. Chr 17 was denatured at 95 °C for 12 min and hybridized at 44 °C for 2 h. After hybridization, appropriate stringency washes (three times at 59 °C) were performed. The HER2 and Chr 17 dinitrophenol-labeled probes were visualized using a rabbit anti-dinitrophenol primary antibody and the ultraVIEW SISH detection kit. The detection kit contains a goat anti-rabbit antibody conjugated to horseradish peroxidase used as the chromogenic enzyme. The chemistry of the SISH reaction, briefly described, is driven by the sequential addition of silver A (silver acetate), silver B (hydroquinone), and silver C (H_2_O_2_). In this study, the silver ions (Ag^+^) were reduced using hydroquinone to metallic silver atoms (Ag). This reaction was fueled by the substrate for horseradish peroxidase, hydrogen peroxide (silver C). The silver precipitate was deposited in the nuclei, and a single copy of the HER2 gene was visualized as a black dot. The specimen was then counterstained with Ventana Hematoxylin II for interpretation via light microscopy.

### 4.6. 2,5-Diphenyl-2H-Tetrazolium Bromide (MTT) Assay

The MTT assay was performed using a thiazolyl blue tetrazolium bromide (MTT; Sigma, St. Louis, MO, USA) colorimetric assay, as previously described [57,58]. Briefly, 1000–3000 cells were seeded in a 96-well plate and incubated overnight. The next day, different concentrations of samfenet (50–5000 µg/mL) were treated and incubated for 72 h at 37 °C. After incubation, the medium was replaced with dimethyl sulfoxide, and MTT solution was added for 30 min. Finally, absorbance was measured at 540 nm using a microplate reader (Multiscan GO Microplate Spectrophotometer, Thermo Fisher Scientific, Waltham, MA, USA) and cell viability was analyzed.

### 4.7. Colony Formation Assay

The colony formation assay was performed using crystal violet colorimetric assay. Between 200 or 300 cells were seeded into 12-well plates and incubated overnight. The next day, effector NK cells were incubated with or without a CD16 blocker for 30 min and then co-cultured with target cancer cells at effector/target (E/T) ratio of 10:1. The co-culture was conducted with or without 400 μg/mL of samfenet for 4 h at 37 °C. The effector cells were then removed, and the target cells were incubated for 10 days. After incubation, the cells were washed with PBS, fixed with 2% paraformaldehyde (PFA) for 10 min at RT, and then the PFA was removed. The cells were stained with 0.2% of crystal violet for 15 min at RT. Finally, colonies were counted, and the % colony formation was analyzed.

### 4.8. Antibody-Dependent Cellular Cytotoxicity Assay

ADCC assay was performed using the CFSE-7AAD cytotoxicity assay. Target cancer cells were incubated with 5 mM of carboxyfluorescein diacetate succinimidyl ester (CFSE; C34554, ThermoFisher Scientific, Waltham, MA, USA) in PBS for 15 min at 37 °C. Cells were washed with PBS and medium, respectively, and cultured overnight. The next day, cells were pretreated with samfenet for 30 min. Simultaneously, effector NK cells were incubated with or without a CD16 blocker for 30 min, and then co-cultured with CFSE-labeled, samfenet-pretreated target cells at effector/target (E/T) ratios of 1:1, 5:1, and 10:1. The co-culture was performed with or without samfenet at a concentration of 1 mg/mL for 4 h at 37 °C. The cells were harvested, washed, and stained with 7-aminoactinomycin D (7-AAD; A1310, ThermoFisher Scientific, Waltham, MA, USA) for 30 min at room temperature. Finally, cells were washed with PBS and analyzed using a CytoFLEX flow cytometer.

### 4.9. Isolation and Ex Vivo Expansion of Human Allogeneic Primary NK Cells

For preliminary in vivo primary NK treatment, human allogeneic primary NK cell isolation and ex vivo expansion procedures were carried out following the previously described method [59]. Human peripheral blood mononuclear cells were collected from a healthy donor via leukapheresis. CD3-CD56+ pNK cells were obtained through CD3 depletion and CD56 positive selection with anti-CD3 (Miltenyi Biotec, Bergisch Gladbach, Germany) and anti-CD56 antibodies (Miltenyi Biotec) using the closed and automated platform, Prodigy (Miltenyi Biotec). The isolated pNK cells were expanded using a GMP-compliant method at CHA Biotech (Seongnam, Republic of Korea). pNK cells were seeded on γ-globulin (Greencross, Yongin, Republic of Korea) and anti-NKp46 (R&D Systems, Minneapolis, MN, USA) coated flasks and cultured in Alys505NK serum-free medium (CSTI, Sendai, Japan) supplemented with 1000 IU/mL recombinant human IL-2 (Novartis, Basel, Switzerland), 50 ng/mL recombinant human interleukin-18 (R&D Systems, Minneapolis, MN, USA), and 5% heat-inactivated autologous plasma. Fresh culture medium was added every 1–3 days, depending on the cell density (2  ×  10^6^ cells/mL). On Day 6, the cells were transferred to a culture bag (NIPRO, Osaka, Japan) and cultured for 14 days. Expanded pNK cells were cryopreserved and thawed following the in vivo infusion schedule.

### 4.10. Establishment of the Patient-Derived Tumor Xenograft Model

Fresh tumor tissues from patients with ovarian cancer were obtained from the CHA Bundang Medical Center, CHA University, Seongnam, Republic of Korea. They were then implanted into the NOD.Cg-PrkdcscidIl2rγtm1Wjl/SzJ (NOD-scid IL2rγnull, NSG) mice, resulting in the growth of a xenograft termed F0-PDTX. The serially passaged PDTXs were called F1, F2, F3, and so on. In this study, we first implanted the F3 PDTX tissue (PDTX 18-4) into the NOD-Prkdc^em1^ mice (SCID/g) and allowed them to grow. The implanted PDTX tissue was isolated, chopped into similar sizes (approximately 50–70 mm^3^), and reimplanted into BALB/C nude mice. These mice were used in drug efficacy assessments.

### 4.11. In Vivo Efficacy Test

Five-week-old BALB/C nude mice were purchased from Gem Biosciences (Cheongju, Korea) and kept in pathogen-free conditions. Mice were acclimatized for one week before the experiments. All animal procedures were performed according to the IACUC-approved protocol of CHA University. As mentioned earlier in the Section 2, using IHC, we confirmed PDTX 18-4 as a HER2-overexpressing PDTX tissue among eight different PDTX tissues.

When the tumor size reached 80–100 mm^3^, the mice were randomly divided into the control (PBS, IP injection, twice weekly), samfenet (10 mg/kg, IP injection, twice weekly), pNK cell (1 × 10^7^ cells, IV injection, twice weekly), and combination (samfenet: 10 mg/kg, IP injection and pNK cells: 1 × 10^7^ cells or NK92-CD16: 5 × 10^6^ cells, IV injections, twice weekly) groups. In this study, a total of six pNK injections were administered due to the limited availability of pNK cells from the supplier. Each group comprised two mice. After 24 days of drug treatment, the mice were euthanized, and the xenografted tumors were excised and preserved for further analysis.

To further validate our in vivo results, we used the NK92-CD16-expressing NK cells because of their similar ADCC effect to that of the pNK cells [60]. As described above, the F3 generation PDTX tissue (PDTX 18-4) was implanted into NOD/SCID mice and subsequently reimplanted into similar-sized tumor tissues in the BALB/C nude mice. Drug efficacy was assessed as previously described. When tumor size reached 80–100 mm^3^, mice were randomly divided into four groups: control, samfenet, NK92-CD16, and combination groups, with a total of eight injections administered (five mice per group). After 32 days of treatment, the mice were euthanized, and the xenografted tumors were excised and preserved for further analysis.

### 4.12. Terminal Deoxynucleotidyl Transferase dUTP Nick End Labeling Assay

TUNEL assay was conducted to evaluate apoptosis in the xenografted PDTX tissues following the manufacturer’s instructions (cat# S7101, Merck KGaA, Darmstadt, Germany). Briefly, after dewaxing and hydration, tissue slides were treated with TUNEL and counterstained with hematoxylin solution for 5 min. TUNEL-positive cells were observed under a light microscope at 400× microscopic fields. ImageJ software version 1.53t was used to quantify positively stained cells.

### 4.13. Public Gene Expression Profiling Datasets of Patients with Serous Ovarian Cancer

To evaluate whether the HER2 mRNA expression level is associated with prognosis in patients with serous ovarian carcinoma, four independent public mRNA expression datasets of serous ovarian carcinoma (GSE 15622, GSE 30161, GSE63885, and TCGA) were analyzed among seven datasets using the online Kaplan–Meier plotter (www.kmplot.com, accessed on 30 June 2024) [61,62]. Affymetrix ID 210930 was selected for HER2, and GeneChip Human Genome U133A (GSE 15622, TCGA) and U133 (GSE 30161, GSE 63885) arrays were used for the datasets. The HER2 mRNA expression level was divided into two groups, HER2-High and HER2-Low, with cutoff set to auto-select the best cutoff. PFS and OS were analyzed based on the HER2 mRNA expression level.

### 4.14. Statistical Analysis

Student’s *t*-test analysis was performed to compare the two groups in the in vitro NK cytotoxicity assay and in vivo experiments, IHC, and TUNEL assay using SPSS version 20 (IBM SPSS Statistics 20, Armonk, NY, USA). PFS was defined as the time from cancer therapy to ovarian cancer progression or the last date at which the patient was known to be free of progression (censoring time), whereas OS was defined as the time from diagnosis to death or the last date at which the patient was known to be alive (censoring time). PFS and OS were calculated using the Kaplan–Meier method. The log-rank test was used to compare PFS and OS between groups. All *p*-values were two-sided, and *p*-values < 0.05 were considered statistically significant.

## Data Availability

All the data from this study are available upon request.

## Supplementary Materials

The following supporting information can be downloaded at https://www.mdpi.com/article/10.3390/ijms252111733/s1.
