# Supplementary material for: Trastuzumab-Mediated Antibody-Dependent Cell-Mediated Cytotoxicity (ADCC) Enhances Natural Killer Cell Cytotoxicity in HER2-Overexpressing Ovarian Cancer
Source: Int J Mol Sci. 2024 Oct 31;25(21):11733. doi: 10.3390/ijms252111733 (PMC11545925; doi:10.3390/ijms252111733)
Supplement: Supplementary file 1 [file ijms-25-11733-s001.zip › ijms-3245345-supplementary.pdf]

International Journal of *Molecular Sciences*

MDPI

Table S1. Table of clinical trials with HER2-expressing ovarian cancer and HER2-targeted therapy. Abbreviation: ORR: overall response rate; RR: response rate; SD: stable disease; PD: progressive disease

Trial name	Cancer type (HER2+)	Status	Phase	Number of patients	Drug	Outcomes	ref
NCT00034281	Solid cancer	Completed	1	16	TAK-165	Not posted	
NCT00433407			Not applicable	12	Trastuzumab	Not posted	
NCT00028535			1	18	Trastuzumab<br>Paclitaxel<br>rIL-12	Not posted	
NCT00004074			1	15	Recombinant interleukin-12<br>ABI-007/carboplatin/trastuzumab	Not posted	
NCT01384253	Intraperitoneal cancer		1	18	^{212}Pb-TCMC-Trastuzumab<br>trastuzumab	Not posted	
NCT03602079	Relapsed/refractory cancer		1/2	49	A166	Best ORR: 59.1%	
	Recurrent ovarian cancer		1	16	^{212}Pb-TCMC-Trastuzumab	6 weeks response<br>SD: 12<br>PD: 4	[1]
NCT00189579	Ovary cancer	Terminated	2	41	Trastuzumab	ORR: 7.3%	
NCT05395052	Solid cancer		1	5	FT536<br>Trastuzumab	Not posted	
NCT04639219	HER2-activating mutation cancer	Active, not recruiting	2	102	Trastuzumab deruxtecan	RR: 29.4%	
NCT02892123	Solid cancer		1	279	Zanidatamab		
NCT03821233			1	174	ZW49		
NCT04660929			1	48	CT-0508 , Pembrolizumab		
NCT04319757	Solid cancer	Recruiting	1	36	ACE1702		
NCT06293898			1	280	BL-M07D1		

NCT04482309			2	468	Trastuzumab deruxtecan		
NCT06003231			2	160	Disitamab vedotin		
NCT04511871			1	15	CCT303-406		
NCT05143970			1	27	IPH5301 and Trastuzumab		
NCT06420973	Ovary cancer		2	54	RC48		
NCT05786716	HER2 amplification or mutation solid cancer		2/3	30	Trastuzumab Pertuzumab		
NCT04828616	Advanced Ovary cancer	Unknown	2	104	DP303c		

References

1. Meredith, R.; Torgue, J.; Shen, S.; Fisher, D. R.; Banaga, E.; Bunch, P.; Morgan, D.; Fan, J.; Straughn, J. M., Jr., Dose escalation and dosimetry of first-in-human α radioimmunotherapy with 212Pb-TCMC-trastuzumab. *J Nucl Med* **2014,** 55, (10), 1636-42.
